# Transmission of an Oxygen Availability Signal at the *Salmonella enterica* Serovar Typhimurium *fis* Promoter

**DOI:** 10.1371/journal.pone.0084382

**Published:** 2013-12-16

**Authors:** Andrew D. S. Cameron, Carsten Kröger, Heather J. Quinn, Isobel K. Scally, Anne J. Daly, Stefani C. Kary, Charles J. Dorman

**Affiliations:** 1 Department of Microbiology, Moyne Institute of Preventive Medicine, School of Genetics and Microbiology, Trinity College Dublin, Dublin, Ireland; 2 Department of Biology, University of Regina, Regina, SK, Canada; University of Manchester, United Kingdom

## Abstract

The nucleoid-associated protein FIS is a global regulator of gene expression and chromosome structure in *Escherichia coli* and *Salmonella enterica*. Despite the importance of FIS for infection and intracellular invasion, very little is known about the regulation of *S. enterica fis* expression. Under standard laboratory growth conditions, *fis* is highly expressed during rapid growth but is then silenced as growth slows. However, if cells are cultured in non-aerated conditions, *fis* expression is sustained during stationary phase. This led us to test whether the redox-sensing transcription factors ArcA and FNR regulate *S. enterica fis*. Deletion of FNR had no detectable effect, whereas deletion of ArcA had the unexpected effect of further elevating *fis* expression in stationary phase. ArcA required RpoS for induction of *fis* expression, suggesting that ArcA indirectly affects *fis* expression. Other putative regulators were found to play diverse roles: FIS acted directly as an auto-repressor (as expected), whereas CRP had little direct effect on *fis* expression. Deleting regions of the *fis* promoter led to the discovery of a novel anaerobically-induced transcription start site (P*fis*-2) upstream of the primary transcription start site (P*fis*-1). Promoter truncation also revealed that the shortest functional *fis* promoter was incapable of sustained expression. Moreover, *fis* expression was observed to correlate directly with DNA supercoiling in non-aerated conditions. Thus, the full-length *S. enterica fis* promoter region may act as a topological switch that is sensitive to stress-induced duplex destabilisation and up-regulates expression in non-aerated conditions.

## Introduction

The factor for inversion stimulation (FIS) is a global regulator of gene expression and chromosome compaction in Gamma-proteobacteria. In *Escherichia coli* and *Salmonella enterica*, transcription of the *fis* gene is very high during rapid growth, consequently FIS is one of the most abundant DNA-binding proteins during exponential growth (50,000 - 100,000 monomers/cell) [1-3]. Transcription of *fis* declines dramatically as cells enter stationary phase, and the FIS protein concentration drops to undetectable levels [1-3]. Control of *fis* expression is best understood in *E. coli*, where the global regulatory proteins CRP, IHF, and FIS modulate only the degree of induction, but none of them is absolutely required for *fis* expression in laboratory culture [4-6]. In their absence, *fis* continues to be induced by nutritional upshift and repressed during stationary phase [4-6]. A major determinant of growth phase-dependent *fis* expression is the GC-rich sequence between the −10 RNA polymerase binding site and the transcription start site, which creates a protein-independent barrier to transcription [7,8]. When DNA becomes highly supercoiled during rapid growth, the topological stress exerted on the DNA double strand facilitates melting of the GC-rich discriminator by RNA polymerase. Thus, DNA supercoiling directly controls *fis* expression by removing a repressive barrier [9]. The discriminator is so-called because it allows RNA polymerase to discriminate between promoters that are subject to the stringent response (like the *fis* promoter) and those that are not [9]. Stringently-regulated promoters respond negatively to the alarmone guanosine tetraphosphate (ppGpp) and to the protein DksA, both of which act via RNA polymerase [10,11].

FIS is an important transcription factor in natural environments where bacteria are starved for nutrients. For example, *S. enterica* serovar Typhimurium (*S*. Typhimurium) requires FIS for pathogenicity gene expression during the initial stages of tissue invasion and later inside the macrophage vacuole [12,13]. Transcriptome analysis has revealed that *S. enterica* serovar Typhi *fis* is induced inside macrophage vacuoles despite nutrient-poor conditions and slow growth [14]. The expression of *fis* in intracellular environments may be explained in part by the recent finding that *S*. Typhimurium *fis* expression is elevated in stationary phase if oxygen availability is reduced, a condition referred to as “sustained expression” because the *fis* promoter remains active in stationary phase [13]. Sustained *fis* expression results in highly elevated FIS protein levels, which contribute directly to increased invasion of epithelial cells in *in vitro* assays [13]. It is superficially similar to the elevated expression seen with genetically altered derivatives of the *E. coli fis* promoter where the initiation nucleotide +1C is changed to either A or G: in these mutants *fis* transcription is elevated in stationary phase [15]. The activity of the native *fis* promoter shadows the fluctuation in the CTP pool of the cell. Our observations of sustained *fis* expression involve the native promoter and the goal of the study presented here was to identify the regulatory mechanisms that link oxygen availability to *fis* expression. 

The redox sensors ArcAB and FNR regulate transitions from aerobic to anaerobic growth, making these proteins prime candidates for regulating the sustained expression of *fis* in low oxygen environments. In the ArcAB two-component system, ArcA is a site-specific DNA binding protein that is activated by phosphorylation when ArcB senses a drop in redox potential [16]. FNR is a site-specific DNA binding protein that becomes active as a transcriptional regulator when its Fe-S cluster is reduced in the absence of oxygen [17]. ArcA and FNR regulate dozens and hundreds of genes, respectively, many of which are required for metabolism in anaerobic conditions, but neither transcription factor has been implicated in controlling *fis* expression. 

In bacteria, DNA supercoiling changes in response to environmental conditions such as nutrient and oxygen availability [10,18]. Because DNA shape influences gene promoter activity, DNA supercoiling is a mechanism for orchestrating global cellular transcription in response to changing conditions [19]. During rapid growth, *E. coli* maintains high levels of DNA supercoiling and this stimulates transcription of *fis* along with other growth phase-dependent promoters, such as rRNA promoters [9,19,20]. Several studies have found that a minimal promoter region containing only the σ^70^ (RpoD) binding site is sufficient for the induction of the *fis* promoter during rapid growth [6,8]; this suggests that DNA supercoiling alone can activate the *fis* promoter in the absence of protein transcription factors, at least in laboratory conditions. 

Genetic analysis revealed that neither the stringent-response-associated alarmone ppGpp nor the DksA protein were essential for the sustained expression of *fis* in stationary phase [21]. We examined the relative contributions made by other protein transcription factors and DNA supercoiling to the expression of *S*. Typhimurium *fis* in the non-aerated growth conditions that up-regulate the *fis* promoter. This led to the discovery of a second transcription start site in the *S*. Typhimurium *fis* promoter, which becomes more active as transcription from the primary transcription start site decreases in the absence of oxygen. Together the results suggest that the full-length *fis* promoter region activates *fis* expression in response to DNA supercoiling in low-aerated conditions. This topological switch may work in concert with multiple transcription start sites and the alternate sigma factor RpoS to integrate environmental and physiological signals at the *S*. Typhimurium *fis* promoter.

## Materials and Methods

### Bacterial strains and reporter gene constructs

*Salmonella enterica* serovar Typhimurium strain SL1344 and mutant derivatives were used for all experiments. Detailed descriptions of strains used in this study are provided in Table S1. Deletion mutants were constructed by the method of Datsenko and Wanner [22] using the recombineering primers listed in Table S2. Mutations were transduced into a fresh SL1344 background by bacteriophage P22 generalized transduction [23] and were confirmed by PCR and DNA sequencing. The pCP20 plasmid, which encodes the FLP recombinase, was used to remove the kanamycin resistance cassette from the *dusB-fis-gfp* ^TCD^::*kan* ^*R*^ construct in the *S*. Typhimurium chromosome as described in [22]. After curing cells of pCP20, loss of antibiotic resistance was confirmed by screening colonies for kanamycin sensitivity using LB-plates containing kanamycin at 50 μg/ml. Construction of SL1344 Δ*crp* was confirmed phenotypically by sugar fermentations tests using peptone broth containing a sugar or sugar alcohol at 0.5% final concentration and bromothymol blue as an indicator. As expected SL1344 Δ*crp* was unable to ferment mannitol, sorbitol or maltose but retained its ability to ferment glucose. 

Promoter truncates were generated by inverse PCR using the primers listed in Table S2 and pZepfisS as template [13]. PCR amplicons were phosphorylated by T4 polynucleotide kinase and then circularised by ligation with T4 DNA ligase, followed by transformation into chemically-competent *E. coli* DH5α. PCR primers targeting the *fis* promoter region included a restriction enzyme site to facilitate confirmation of correct ligation products.

### Culture growth

Well-aerated conditions were achieved by using 10 ml of LB (1% tryptone, 0.5% yeast extract, 0.5% NaCl) in a 250 ml glass conical flask, whereas non-aerated conditions used the same culture volume in a glass tube with an interior diameter 14 mm, as in [13]. All containers were loosely capped to allow air flow, and shaken at 200 RPM at 37 °C. Cultures were started by diluting cells 1/100 from well-aerated overnight cultures and grown for 22 hours to ensure that they had reached steady-state stationary phase before sampling. Anaerobic shock was administered by transferring 15 ml of well-aerated exponentially-growing cell culture into a 15 ml centrifuge tube, capping tightly, and incubating without agitation for 30 min at 37 °C. When required, antibiotics were used at the following final concentrations: carbenicillin 100 µg/ml, chloramphenicol 20 µg/ml, and kanamycin 50 µg/ml.

### Flow cytometry and quantitative PCR

GFP levels in cells expressing *fis-gfp* ^TCD^ and P*fis*-*gfp*+ were quantified using flow cytometry as follows: 2-30 µl of culture was fixed in 700 µl of freshly prepared phosphate buffered saline containing 2% formaldehyde. Fixed samples were stored overnight in the dark at 4 °C. The median fluorescence of 20,000 cells/sample was measured on a Dako CyAn ADP flow cytometer (PMT voltage 800-875 V). To quantify transcript abundance, RNA was isolated using TRIzol as described earlier [24]. Quantitative PCR was conducted as in [25] using primers listed in Table S2. 

### DNA supercoiling analysis

Plasmids were isolated from cultures using the HiYield Plasmid Mini Kit (RBC Bioscience). All electrophoresis was conducted in 27-cm-long 1% agarose gels with 2x Tris Borate EDTA (TBE) as gel and running buffer. Approximately 1 µg of plasmid DNA (8–15 µl) was loaded on a gel using 4 µl of loading buffer (80% glycerol, 0.5 mg/ml bromophenol blue). The gel and buffer contained 2.5 µg/ml chloroquine, and electrophoresis was performed at 3 V/cm for 16 hours. To remove the chloroquine after electrophoresis, gels were washed by gentle rocking in large volumes of tap water for at least 3 hours; the wash water was replaced every 20–30 minutes. After washing, gels were stained by gentle rocking in water containing ethidium bromide (1 µg/ml) for at least 1 hour, then washed briefly in water, and plasmid topoisomers were visualized with UV light. Analyses of topoisomer distributions were conducted as in [26].

WebSIDD [27] was used to predict how DNA supercoiling affects stability of the *S*. Typhimurium and *E. coli fis* promoter regions. WebSIDD uses a default 5 kbp window that slides by 500 bp. Thus the stability of each base pair, G(x), is the average of 10 calculations, where the influence of proximal bases is weighted according to how close the base pair is to the center of the sliding window. 

### 5′ RACE and western blot analysis

5′ RACE was conducted as in [28] using the primer Pfis.expTSS.RT.Rev to reverse transcribe *dusB-fis* transcripts. PCR products were cloned into the linearized vector pJET (Fermentas) and transformed into *E. coli* strain XL-1 and at least 5 clones were DNA sequenced. Western blot analysis was conducted as in [25] using the *E. coli* σ^S^ (RpoS) monoclonal antibody (Neoclone) diluted 1:5,000.

## Results

### Comparison of *S*. Typhimurium and *E. coli fis* promoter regions

**Figure 1:**
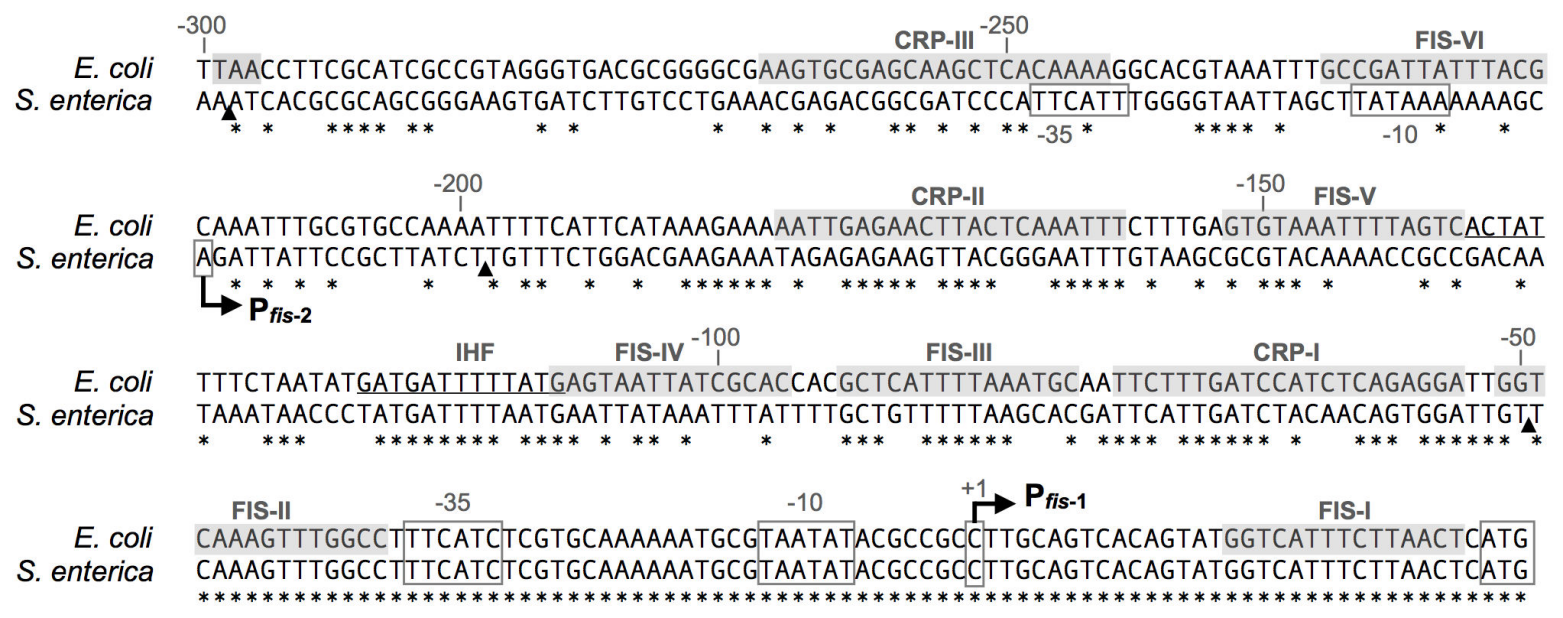
Gapless sequence alignment of the *E. coli* and *S*. Typhimurium *fis* promoter regions. CRP and FIS (grey boxes) and IHF (underline) binding sites identified in *E. coli* are shown [5,6,40]. Promoter elements, transcription start sites and *dusB* translation initiation codons are boxed. The angled arrows show the locations of P*fis*-1 (both species) and P*fis*-2 (*S*. Typhimurium). The locations of promoter truncations in *S*. Typhimurium are indicated by black triangles.

The regulation of *fis* expression has been studied primarily in *E. coli*. As a first step to identify the regulatory elements that control *S*. Typhimurium *fis* expression, the *S*. Typhimurium *fis* region was aligned with the same region in *E. coli*. The bicistronic *dusB*-*fis* operon encodes FIS in both species. Because dusB has attracted very little research attention, the upstream promoter region (P*fis*) bears the *fis* name. The *dusB* and *fis* open reading frames have 92% and 98% nucleotide identity, respectively, between *E. coli* and *S*. Typhimurium, indicating that very little change has occurred in these genes since divergence of the *Escherichia* and *Salmonella* genera. As has been noted previously, the region containing the primary transcription start site is identical between *E. coli* and *S*. Typhimurium [20], but there is significant sequence divergence upstream of position −49 (Figure 1). In E. coli there is an intergenic space of 296 bp between the *dusB-fis* transcription start site and the stop codon of the upstream gene, *prmA*. In *S*. Typhimurium, the *prmA* stop codon is 628 bp upstream, suggesting that >300 bp of non-coding DNA was inserted upstream of the ancestral promoter region. Alignment of DNA sequence from other *Enterobacteriaceae* species confirms that the ancestral promoter region was likely around 300 bp (not shown), making Salmonella unusual in the *Enterobacteriaceae* family. 

To test whether the full-length intergenic region contributes to regulation, the *gfp* ^TCD^ reporter gene was fused to the *S*. Typhimurium *dusB-fis* operon in the native chromosomal location. Because FIS is autoregulatory, we avoided creating a *fis* null mutant by inserting *gfp* ^TCD^ downstream of the *fis* open reading frame, but upstream of a predicted transcriptional terminator. The kanamycin resistance marker linked to *gfp* ^TCD^ was subsequently excised to prevent the kan promoter from influencing *fis* expression. This chromosome-based reporter differs from the study by Ó Cróinín and Dorman that first identified sustained *fis* expression [13]. Ó Cróinín and Dorman used a plasmid-based reporter gene fusion containing the *fis* promoter region up to -298 bp, thus omitting the 330 bp of upstream non-coding sequence. 

**Figure 2:**
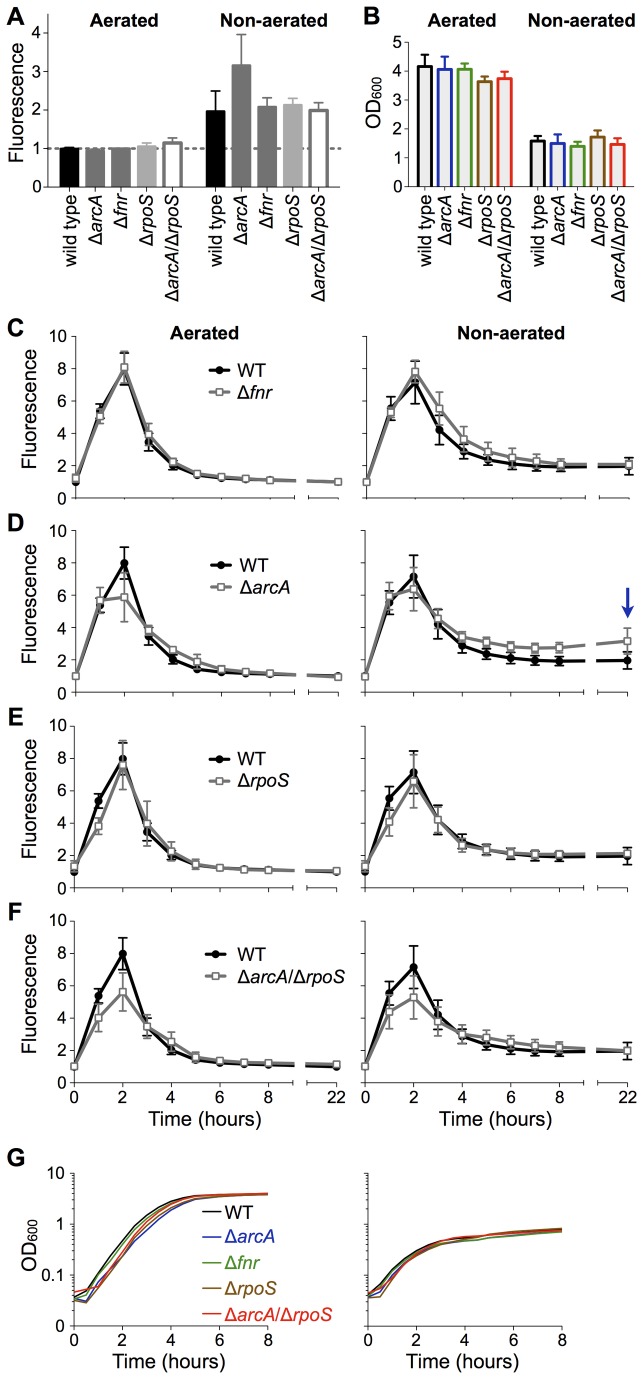
Effects of transcription factor mutations on *fis* expression in aerated and non-aerated conditions. A) Column graph comparing *dusB-fis*::*gfp* ^TCD^ expression at 22 hours. All data are expressed relative to wild type at 22 hours in well-aerated conditions (dashed line). **B**) Average and standard deviation of four replicate culture densities after 22 hours. C-F) Time courses of *dusB-fis::gfp* ^TCD^ expression in *S*. Typhimurium wild type and Δ*fnr* (C), Δ*arcA* (D), Δ*rpoS* (E), and Δ*arcA*/Δ*rpoS* (F) mutants. The blue arrow in D indicates the increased sustained expression observed in the Δ*arcA* mutant after 22 hours in non-aerated conditions. **A**, **C**-**F**) Mean and standard deviation of GFP fluorescence from three or four biological replicates are plotted in arbitrary units. All data are expressed relative to wild type levels at time point 0 in well-aerated conditions. The same wild type data are presented in panels C-F. **G**) Growth dynamics of strains in aerated and non-aerated conditions. Smoothed curves were generated by GraphPad Prism 5.0d from the average of four or more replicate growth curves.

In stationary phase cultures the *dusB-fis-gfp* ^TCD^ construct demonstrated elevated expression in non-aerated conditions compared to aerated conditions (Figure 2A). This modest two-fold increase in expression corresponds to a dramatic rise in FIS protein level, which causes *S*. Typhimurium to become more invasive in a *fis*-dependent manner [13]. Overall, the *dusB-fis-gfp* ^TCD^ chromosomal fusion exhibited the classic expression pattern of strong induction in exponential growth in both well-aerated and non-aerated conditions (Figure 2C), followed by a rapid decrease in expression as growth slowed. Two-fold higher fluorescence was sustained from 3 to 22 hours of growth in non-aerated compared to aerated conditions (Figure 2A & C). The two-fold difference we observed with the *dusB-fis-gfp* ^TCD^ construct was less than the increase seen previously in the plasmid-based reporter system [13], and quantitative PCR confirmed that our chromosomal construct is an accurate reporter of native *fis* gene expression (Figure S1). The reduced intensity of sustained expression in the chromosome *gfp* fusion compared to the plasmid-borne reporter indicates that the plasmid system is a reliable but exaggerated reporter of sustained *fis* expression in non-aerated conditions.

### ArcA is a repressor of *S*. Typhimurium *fis*

The redox sensors ArcAB and FNR are transcriptional activators in low oxygen conditions, making them prime candidates for activating sustained *fis* expression. Deletion of *fnr* had no detectable effect on *fis* expression or on cell growth (Figure 2B, C & G). Surprisingly, deletion of *arcA* caused an increase in *fis* expression in non-aerated stationary phase (Figure 2A & D), implicating *arcA* as a repressor of sustained *fis* expression. We found that although the Δ*arcA* mutant had a slightly prolonged lag phase in fresh medium, its growth rate and final cell densities were very similar to wild type cells in both aerated and non-aerated conditions (Figure 2B & G). Thus, the effect of the Δ*arcA* mutation on *fis* expression is not due to altered growth in non-aerated conditions.

In *E. coli* ArcA is a known repressor of *rpoS* transcription [29], and RpoS has been proposed to repress *fis* expression in *S*. Typhimurium [13], thus an *S*. Typhimurium Δ*arcA* mutant would be predicted to have reduced *fis* expression due to up-regulation of *rpoS*. This led us to test whether ArcA may act through *rpoS* in *S*. Typhimurium, perhaps by up-regulating *rpoS* expression as opposed to repressing it as in *E. coli*. First though we were surprised to discover that the chromosomal *dusB-fis-gfp* ^TCD^ fusion was not up-regulated in the Δ*rpoS* mutant (Figure 2E), which contrasts with the previously observed strong activating effect of the Δ*rpoS* mutation on the plasmid-based P*fis*::*gfp* fusion [13]. Quantitative PCR confirmed that the *dusB-fis-gfp* ^TCD^ fusion is a reliable reporter of *fis* expression in non-aerated conditions (Figure S1). The Δ*rpoS* mutant showed an extended lag phase, but unlike the Δ*arcA* mutant it did not achieve wild type densities in aerated culture after 22 hours; instead it grew slightly better than wild type in non-aerated conditions (Figure 2B & G). Neither of these minor growth phenotypes had a detectable effect on *fis* expression. 

To further address whether ArcA may act indirectly on *fis* through RpoS function, we constructed a Δ*arcA*/Δ*rpoS* double mutant. In aerated and non-aerated growth conditions, the Δ*arcA*/Δ*rpoS* double mutant showed consistently reduced *fis* expression during exponential growth (Figure 2F), unlike either single mutant. Finding an exacerbating effect of combining the two mutations suggests that ArcA acts separately from RpoS to influence *fis* expression in exponential growth conditions. During stationary phase in non-aerated conditions, the Δ*rpoS* mutation nullified the effect of the Δ*arcA* mutation of enhancing sustained *fis* expression (Figure 2A & F), implicating RpoS as a mediator of ArcA’s effect on *fis* expression. 

### The minimal *fis* promoter is deficient in sustained expression

**Figure 3:**
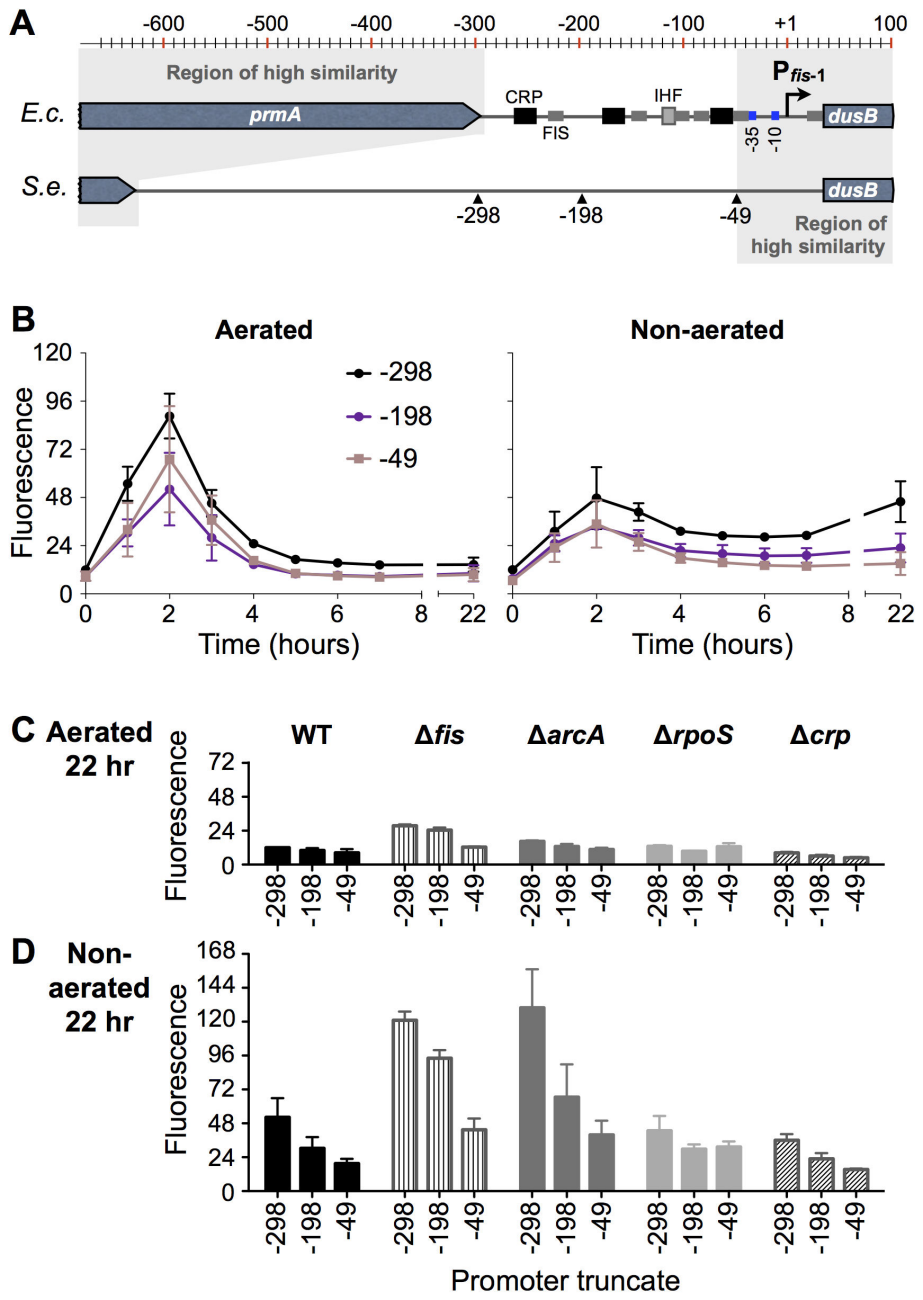
Defining functional regions and transcription factor input at the *fis* promoter. A) Schematic of the *E. coli* and *S*. Typhimurium intergenic regions. The locations of truncations in *S*. Typhimurium are indicated. B) Time courses of expression of plasmid-borne P*fis*::*gfp* promoter truncates in aerated and non-aerated *S*. Typhimurium cultures. C and D) Expression of promoter truncates in *S*. Typhimurium transcription factor mutant backgrounds at 22 hours. In B-D, mean and standard deviation of GFP fluorescence from three or more biological replicates are plotted in arbitrary units that are reported relative to the chromosomal *dusB-fis::gfp* ^TCD^ fluorescence output presented in Figure 2.

Because neither FNR nor ArcA was required for sustained *fis* expression in non-aerated conditions, we created a series of *fis* promoter (P*fis*) truncates to identify promoter regions required for sustained expression. Three truncates were constructed in a plasmid-based *gfp* reporter system. The amplification of *fis* expression profiles by the plasmid system provided two experimental advantages: first, the ability to create truncated promoters, and second, improved sensitivity to detect subtle changes in gene expression. Unfortunately, we were unable to clone the full intergenic region from *S*. Typhimurium, suggesting that an unidentified element upstream of position −300 is toxic to cells when present in multicopy. The construct P*fis*(−298) contained a promoter region identical in length to the *E. coli* intergenic region (Figure 3A), which is the same *S*. Typhimurium promoter region used previously to study sustained expression [13]. The medium length construct P*fis*(−198) removed sequence that in *E. coli* contributes to regulation (Figure 1). The shortest construct, P*fis*(−49), preserved only the region of identity between *S*. Typhimurium and *E. coli*. 

All three constructs demonstrated the classic peak in expression during exponential growth. However, P*fis*(−198) and P*fis*(−49) showed reduced overall expression levels at all stages of growth in both aerated and non-aerated conditions (Figure 3B), indicating that the full 300-bp upstream region contributes to activation. Furthermore, the minimal RpoD-driven promoter, P*fis*(−49), was sufficient for strong induction during exponential growth in *S*. Typhimurium, as reported in *E. coli* [6,8]. In non-aerated conditions, sustained *fis* expression was lower in P*fis*(−198) and was even further reduced in P*fis*(−49) (Figure 3B), indicating that one or more regulatory elements upstream contribute to sustained expression. 

### Transcription factor control of *S*. Typhimurium *fis* expression

To determine which transcription factors contribute to sustained *fis* expression and to identify regions of the *fis* promoter required for transcription factor function, P*fis* truncates were tested in several mutant backgrounds. In *E. coli*, FIS represses its own promoter by binding the FIS-I and FIS-II binding sites that flank the transcription start site [4,6], and these sites are perfectly conserved between the *E. coli* and *S*. Typhimurium *fis* promoters (Figure 1). Up-regulation of the *S*. Typhimurium *fis* promoter in the Δ*fis* mutant confirmed that FIS represses its own promoter, and this repression occurs in both aerated and non-aerated conditions (Figure 3C & D). The FIS-I and FIS-II sites are intact in the shortest construct, P*fis*(−49), allowing FIS to repress each of the three promoter truncates. 

All three lengths of P*fis* showed elevated expression in the Δ*arcA* mutant in non-aerated conditions (Figure 3D). Elevated expression regardless of promoter length supports a model in which ArcA has an indirect influence on *fis* during stationary phase, consistent with the absence of a predicted ArcA site in the *S*. Typhimurium *fis* promoter. *fis* expression was slightly elevated in aerated conditions (Figure 3C), which was not detected using the chromosomal system — again indicating that the plasmid-borne system enhances or exaggerates *fis* promoter activity. 

The Δ*rpoS* mutation did not have a detectable effect on the P*fis*(−298) and P*fis*(−198) truncates, as observed above with the *dusB-fis-gfp* ^TCD^ chromosomal fusion (Figure 3C & D). However, P*fis*(−49) revealed that RpoS can exert a mild repressive effect in the absence of upstream *fis* promoter DNA, both in aerated and non-aerated conditions.

CRP is a global-acting transcription factor that directly regulates FIS expression in *E. coli* [4]. We found that deletion of *crp* caused a slight reduction in *fis* expression from all three promoter truncates in both aerated and non-aerated conditions (Figure 3C & D). The absence of a DNA site matching the CRP binding site consensus (Figure 1) suggest that CRP plays an indirect regulatory role at the *fis* promoter in *S*. Typhimurium. 

### The *S*. Typhimurium *fis* regulatory region has a second, anaerobically-induced promoter

**Figure 4:**
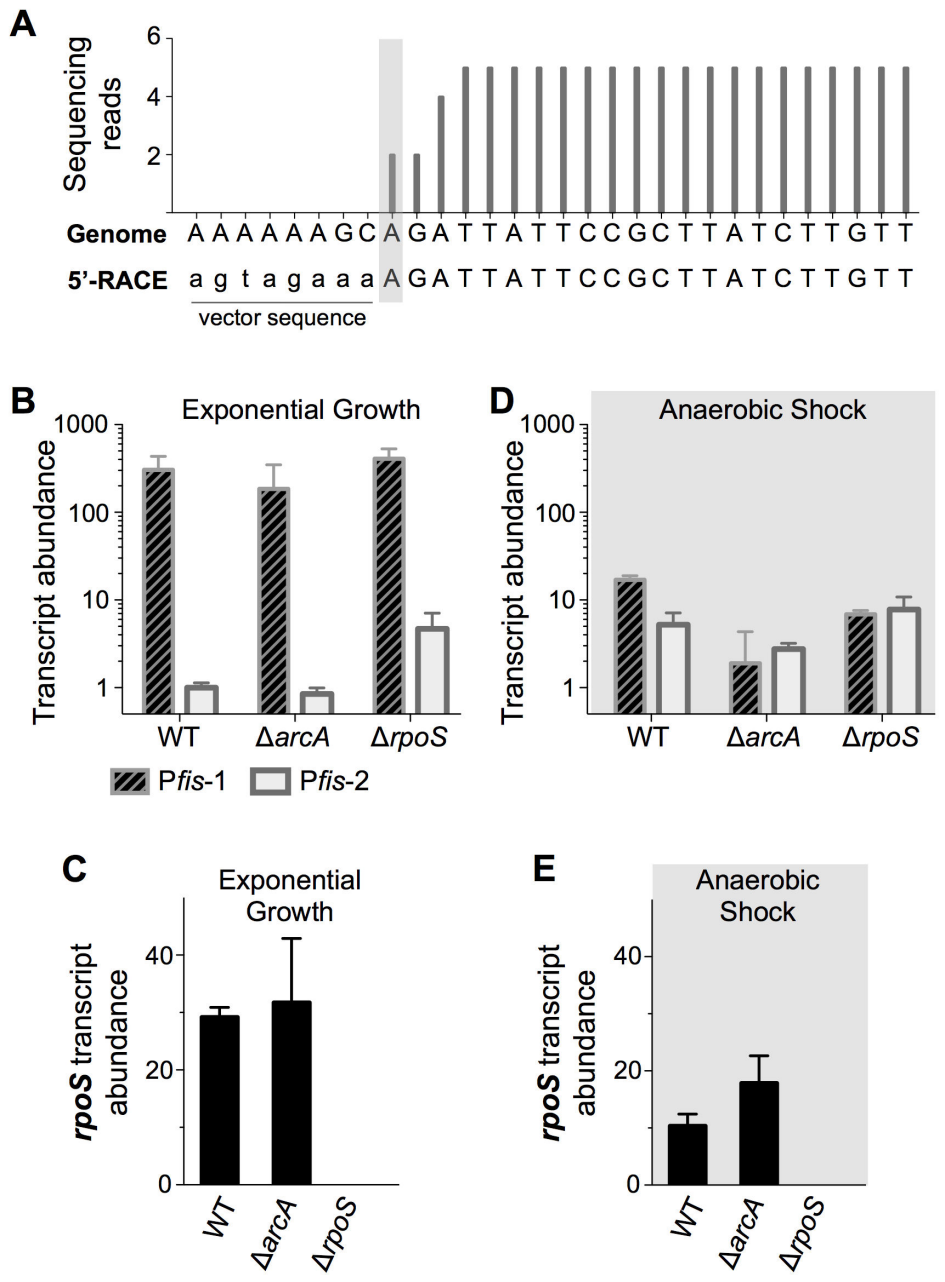
Characterisation of *S*. Typhimurium Pfis-2. A) RNA-seq and 5′-RACE identification of the P*fis*-2 transcription start site. The number of sequencing reads in RNA-seq analysis of an early stationary phase *S*. Typhimurium culture is aligned with DNA sequencing results from 5′-RACE analysis of the same RNA sample (RNA-seq data available in [24]). B) Quantitative PCR measurement of transcripts originating from P*fis*-1 and P*fis*-2 during exponential growth. C) Quantitative PCR measurement of *rpoS* expression. D) Quantitative PCR measurement of transcripts originating from P*fis*-1 and P*fis*-2 during anaerobic shock. E) Quantitative PCR measurement of *rpoS* expression. In **B-E**, the mean and standard deviation from 3 to 5 biological replicates are plotted.

The reduction in *fis* expression caused by progressive truncation of the *fis* promoter could be explained by removal of one or more upstream transcription start sites (TSS). Thus, we used recently published RNA-seq data [24] to search for evidence of transcripts originating upstream of the primary TSS, which revealed a putative TSS at position −216 relative to the primary TSS (Figure 1). 5′-RACE confirmed the existence of the second TSS (Figure 4A), which we named P*fis*-2 to distinguish it from the gene proximal TSS, called P*fis*-1. P*fis*-2 was not included in Kröger et al. [24] because it did not pass the conservative threshold used in that study. 

To determine the activity of P*fis*-2 in laboratory culture, quantitative PCR was used to distinguish between transcripts originating from P*fis*-1 and P*fis*-2. During exponential growth, less than one percent of *dusB-fis* transcripts originated at P*fis*-2 (Figure 4B). Transcription from P*fis*-2 was up-regulated two-fold in non-aerated stationary phase cultures, but this accounted for only 7±1% of total *fis* transcripts, indicating that P*fis*-2 is not responsible for sustained *fis* expression. Transcription was moderately reduced at both P*fis*-1 and P*fis*-2 in the Δ*arcA* mutant (Figure 4B), suggesting that ArcA causes mild activation of *fis* expression during exponential growth. Deletion of *rpoS* revealed RpoS to be a weak repressor of P*fis*-1 and a stronger repressor of P*fis*-2 during exponential growth (Figure 4B). 

To further test whether ArcA acts indirectly through RpoS by repressing *rpoS* transcription, we measured *rpoS* transcript abundance. No significant difference was detected in *rpoS* transcript levels between wild type and Δ*arcA* mutant cells in exponential growth (Figure 4C). RpoS is controlled primarily by post-transcriptional mechanisms; unfortunately attempts to quantify RpoS protein levels in the wild type and Δ*arcA* mutant in exponential growth were unsuccessful because RpoS protein levels were below the detection limit of western blot assays. 

Two-fold up-regulation of P*fis*-2 in non-aerated growth conditions led us to test the effects of more severe anaerobic shock, a condition that, like non-aerated growth, may imitate conditions encountered by *S*. Typhimurium in the intestine and in intracellular environments. Exponentially growing cells were transferred to sealed tubes to induce anaerobic shock, and this caused a significant up-regulation of P*fis*-2 concomitant with 18-fold repression of P*fis*-1 (Figure 4D). In these conditions, deletion of *arcA* caused a decrease in P*fis*-1 and P*fis*-2 activity, consistent with the decrease in exponential phase *fis* expression seen in Figure 2D. To once again test whether the ArcA effect might arise through RpoS activity, *rpoS* expression was measured in anaerobic shock. ArcA was found to exert a repressive effect on *rpoS* as revealed by higher *rpoS* transcript levels in the Δ*arcA* mutant (Figure 4E). Although higher levels of RpoS protein in the Δ*arcA* mutant could explain the repression of P*fis*-2 observed in Figure 4C, the Δ*rpoS* mutant showed wild type levels of P*fis*-2 expression during anaerobic shock (Figure 4D). The absence of an explicit RpoS effect in aerated, non-aerated, and anaerobic conditions (Figures 2E, 4C & D), suggests a model in which RpoS represses *fis* expression only when RpoS protein levels are unusually high, as in a Δ*arcA* mutant. 

### DNA supercoiling control of *S*. Typhimurium *fis* expression

DNA supercoiling is a key driver of *fis* expression [9], raising the intriguing question of whether DNA supercoiling and the *fis* promoter respond to small incremental changes in oxygen availability. Conversely, there may be specific oxygen concentrations at which regulatory control undergoes a transition that facilitates sustained *fis* expression in non-aerated conditions. To assess the relationship between oxygen availability, DNA supercoiling, and *fis* promoter activity, aeration was adjusted by growing cells in tubes with increasing culture volumes. DNA supercoiling and *fis* expression were measured in parallel cultures containing either pUC18 or the P*fis*(-298) reporter plasmid, respectively. The small, high copy number plasmid pUC18 (2,686 bp) was ideal for quantifying supercoiling levels, whereas the larger (6,424 bp) and low copy number reporter plasmid pZec-Pfis was less reliable for resolution of topoisomers; nevertheless, we did find that pZec-Pfis had the same topological responses as pUC, as was previously confirmed for pZec [26]. In the present study, wild type cells carrying pUC18 were assayed in parallel with strains having either the plasmid-borne P*fis*(-298) reporter or the chromosomal *dusB-fis-gfp* ^TCD^ reporter. 

**Figure 5:**
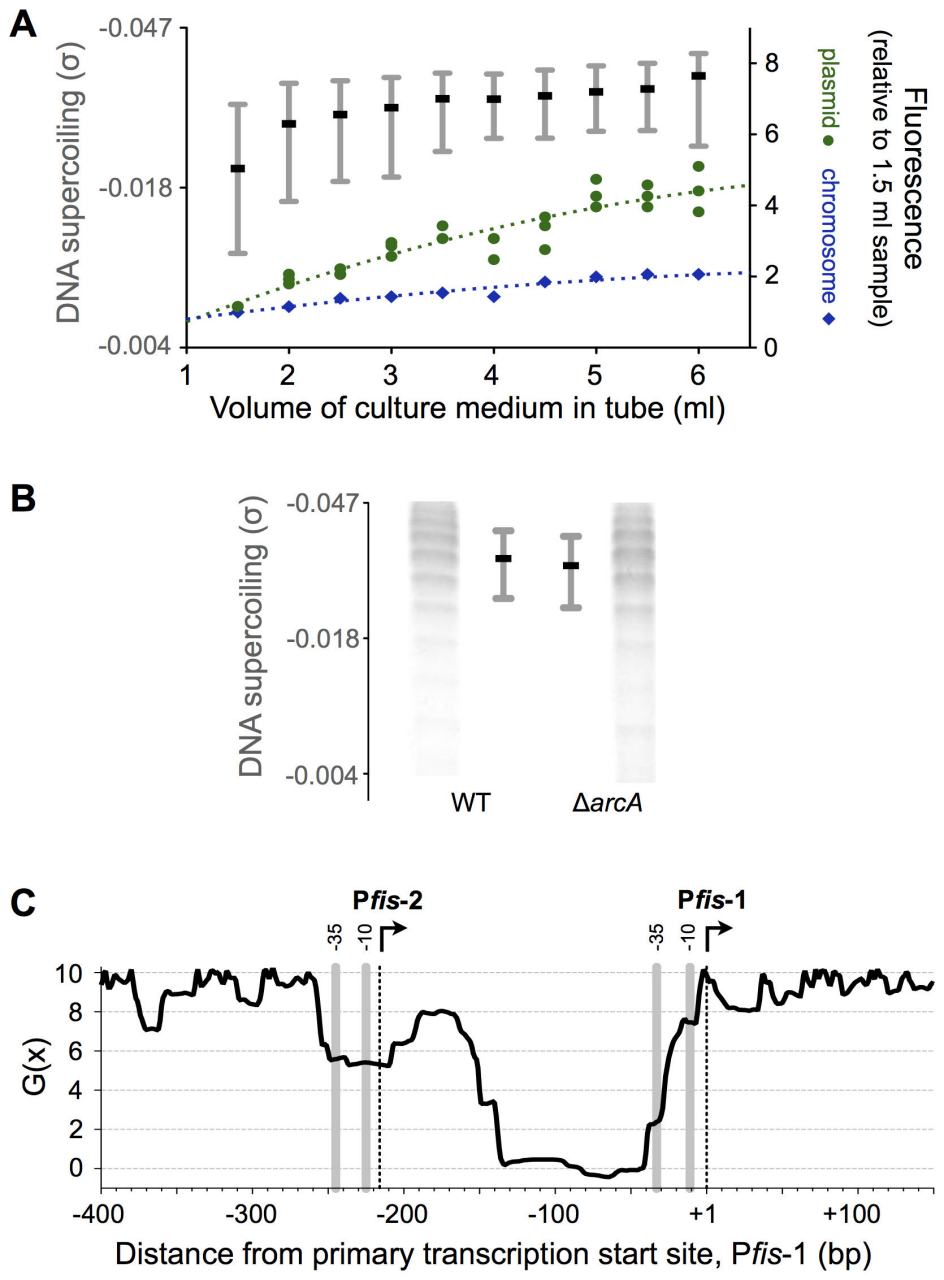
DNA supercoiling control of *fis* expression. A) Median and interquartile ranges of DNA supercoiling from four biological replicates, plotted as in Figure 4E. Fluorescence data from P*fis*(-298) (green circles) and *dusB-fis::gfp* ^TCD^ (blue diamonds) for each biological replicate is plotted on the right y-axis; units as in Figure 2. For both the DNA supercoiling and gene expression measurements, wild type cells were grown to stationary phase cells in the indicated volume of culture medium. The dashed lines are nonlinear curves fit to the expression data, with goodness-of-fit R^2^ >0.85 in both cases. The degree of DNA supercoiling (σ) was determined by measuring the migration of topoisomers relative to fully relaxed DNA in chloroquine gels; each topoisomer represents a change of 1 in the linking number. B) DNA supercoiling states in *S*. Typhimurium wildtype and Δ*arcA* mutant cells at 22 hours in non-aerated conditions. Medians (black bar) and interquartile ranges of pUC18 topoisomer distributions in stationary phase in well-aerated cultures. For each strain, the average interquartile range from four biological replicates is plotted. C) SIDD profile of the *fis* promoter region. The energy required for DNA strand separation at a base pair, G(x), is a function of adjacent and distant DNA sequence [30], and G(x) values below 10 indicate positions prone to SIDD. G(x) values for linear DNA were calculated by WebSIDD [27] using 3,500 bp of chromosomal DNA sequence on either side of the *dusB* start codon (7,000 bp total); only the 550 bp region containing P*fis*-1 and P*fis*-2 is shown.

A continuous increase in DNA supercoiling was observed as aeration decreased due to increased culture volume (Figure 5A). In these same conditions P*fis* expression demonstrated a gradual increase with decreased aeration. Increased expression was observed with both the plasmid-borne and chromosomal reporters, but as expected was more pronounced in the plasmid system. P*fis* expression was the same in 6 ml and the 10 ml “non-aerated” condition used in the experiments above (compare Figures 3D and 5A); thus cells appear to be severely oxygen limited in volumes above 5 ml in standard culture tubes. These results suggest that changes in DNA supercoiling during non-aerated growth may be involved in sustained *fis* expression.

The correlation between DNA supercoiling and sustained *fis* expression in non-aerated cultures prompted us to test whether increased *fis* expression in the ∆*arcA* mutant might also correlate with an increase in DNA supercoiling in this genetic background. DNA supercoiling was not elevated in the Δ*arcA* mutant (Figure 5B).

DNA supercoiling reduces the amount of energy required for transcription initiation by exerting torsional stress on the DNA double helix, which weakens base pairing and facilitates melting; this is referred to as stress-induced duplex destabilization (SIDD) [30]. Because DNA supercoiling stimulates *fis* expression we predicted that the *fis* promoter region would have a SIDD profile indicative of significant stress-induced denaturation, which may be focused near P*fis*-1 and P*fis*-2. Figure 5C plots the predicted stability of the *fis* promoter at the supercoiling level observed both in exponential growth and in non-aerated stationary phase (superhelix density around -0.05). This analysis suggests that the *S*. Typhimurium *fis* promoter region is particularly prone to destabilization and melting when DNA is highly supercoiled. The region immediately upstream of P*fis*-1 (position −10 to −160) is predicted to be extremely destabilized, with a second smaller region of destabilization surrounding P*fis*-2 (position −190 to −260). This very strong SIDD profile raises the possibility that DNA supercoiling alone is able to activate transcription. Further, the observed SIDD profile helps explain why removal of the most destabilized region makes the P*fis*(−49) truncate deficient in sustained expression.

## Discussion

FIS is a global regulator of gene expression, but its abundance in the cell fluctuates dramatically depending on growth phase. During periods of rapid growth in laboratory conditions the *fis* promoter is highly expressed, which accounts for the high levels of FIS protein during exponential growth. Activation of the *fis* promoter during rapid growth relies on highly supercoiled DNA and RpoD [9]. It is possible then that the *fis* promoter is inactive in stationary phase because DNA is in a more relaxed state and because effective concentrations of RpoD are reduced due to competition with RpoS [31]. A reduction in oxygen availability during stationary phase causes DNA to become highly supercoiled in both *S*. Typhimurium and *E. coli* [26,32,33]. Thus a simple model predicts that *fis* will be expressed in oxygen-limited conditions, and our results suggest that DNA supercoiling may be an important contributor to sustained *fis* expression. Further, our findings fit very well with the recent observation that *E. coli fis* expression is much more dependent on global cellular physiology than on the direct activity of transcription factors [34]; we suspect that the regulatory influence of “cellular physiology” posited by the authors is due largely to changes in DNA supercoiling, which they did not test.

The observed reduction in *fis* expression with progressive truncation of the promoter region suggests that the entire promoter region functions as a topological switch, and that the switch loses potency when shortened. DNA supercoiling appears to sit atop the regulatory hierarchy because promoter truncation had the same effect of reducing transcriptional output in each of the *fis*, *arcA*, *rpoS*, and *crp* regulatory mutant strains. A topological switch mechanism is further supported by the prediction of a long destabilised region upstream of the GC-rich discriminator. During rapid growth, the GC-rich discriminator is readily melted and transcription proceeds at a high rate even with a very short promoter region, as in the P*fis*(−49) truncate. However, our data suggest that during the lower energy state of stationary phase, discriminator melting requires a long destabilised region to focus stress-induced melting at the *fis* promoter. The *fis* promoter is particularly striking for its large dynamic range, from very strong to silent. This makes it an ideal model for understanding how DNA supercoiling can be a dominant force in transcriptional control.

How ArcA and RpoS influence *fis* expression remains enigmatic. Both proteins are global regulators, and the pleiotropic effects of mutating global regulators can make it difficult to distinguish direct from indirect mechanisms of gene regulation. The simplest explanation is that ArcA indirectly influences *fis* expression through its activity as a repressor of *rpoS* expression. The expression of *fis* and rpoS is negatively correlated, the former being elevated in exponential growth and the latter in stationary phase, therefore it is unsurprising that FIS and RpoS are antagonistic at some gene promoters. For example, both proteins influence DNA supercoiling; FIS represses *gyrB* [35], whereas RpoS transcribes *gyrB* [36]. The interplay between these two regulators is particularly intriguing in conditions that promote *fis* expression during stationary phase, when RpoS is most active. 

RpoS functions as a non-traditional repressor by (1) competing with RpoD for access to core RNA polymerase and (2) by competing with RpoD for DNA-binding sites because RpoD and RpoS bind very similar DNA sequences. RpoD depends on higher levels of DNA supercoiling than RpoS to initiate transcription [37]. For this reason, promoters can be differentially regulated by RpoD and RpoS through a mechanism in which relaxation of DNA supercoiling causes a transition from RpoD binding to RpoS binding. The *dps* promoter may present a useful model for understanding *fis* regulation by RpoS. Dps is a nucleoid-associated protein with an expression profile that is the opposite of FIS; it is absent during exponential phase growth but becomes highly abundant in stationary phase [3]. During exponential growth, RpoD and FIS bind together and remain locked at the *dps* promoter, thus preventing RpoS from accessing the −35 and −10 elements [38]. When FIS levels decrease as growth slows, RpoS gains access to the *dps* promoter and transcription is up-regulated. It may be that during stationary phase RpoS is able to gain access to and initiate transcription at the otherwise RpoD-driven *fis* promoter. Thus, up-regulation of *rpoS* in the Δ*arcA* mutant causes an elevation in sustained *fis* expression because of elevated RpoS activity at the *fis* promoter.

In *E. coli*, at least five transcription start sites have been detected in the *fis* promoter region [5], with the highly conserved start site (P*fis*-1 in Figure 1) being chiefly responsible for *fis* expression in laboratory conditions [39]. We have identified a novel transcription start site, P*fis*-2, in *S*. Typhimurium, bringing to two the number of characterised start sites in the *S*. Typhimurium *dusB-fis* operon. P*fis*-2 is up-regulated in anaerobic shock and even overtakes P*fis*-1 as the primary source of transcript in the absence of ArcA or RpoS. Anaerobic shock is particularly relevant to the study of *S*. Typhimurium pathogenesis, thus we are currently investigating whether P*fis*-2 plays a significant role in infection systems. 

## Supporting Information

Figure S1 **Quantitative PCR measurement of *fis* transcript in mutants.** Total (P*fis*-1 plus P*fis*-2) *fis* transcript abundance, expressed relative to wild type at 22 hours in well-aerated conditions.
(TIFF)

Table S1 **Bacterial strains and plasmids used in this study.**
(DOCX)

Table S2 **Oligonucleotide primers used in this study.**
(DOCX)

## References

[1] Ball CA, Osuna R, Ferguson KC, Johnson RC (1992) Dramatic changes in Fis levels upon nutrient upshift in *Escherichia* *coli* . J Bacteriol 174: 8043–8056. PubMed: 1459953. 1459953 10.1128/jb.174.24.8043-8056.1992 PMC207543

[2] Osuna R, Lienau D, Hughes KT, Johnson RC (1995) Sequence, regulation, and functions of *fis* in *Salmonella* *typhimurium* . J Bacteriol 177: 2021–2032. PubMed: 7536730. 7536730 10.1128/jb.177.8.2021-2032.1995 PMC176845

[3] Ali Azam T, Iwata A, Nishimura A, Ueda S, Ishihama A (1999) Growth phase-dependent variation in protein composition of the *Escherichia* *coli* nucleoid. J Bacteriol 181: 6361–6370. PubMed: 10515926. 10515926 10.1128/jb.181.20.6361-6370.1999 PMC103771

[4] Nasser W, Schneider R, Travers A, Muskhelishvili G (2001) CRP modulates *fis* transcription by alternate formation of activating and repressing nucleoprotein complexes. J Biol Chem 276: 17878–17886. doi:10.1074/jbc.M100632200. PubMed: 11279109. 11279109

[5] Nasser W, Rochman M, Muskhelishvili G (2002) Transcriptional regulation of *fis* operon involves a module of multiple coupled promoters. EMBO J 21: 715–724. doi:10.1093/emboj/21.4.715. PubMed: 11847119. 11847119 PMC125868

[6] Pratt TS, Steiner T, Feldman LS, Walker KA, Osuna R (1997) Deletion analysis of the *fis* promoter region in *Escherichia* *coli*: antagonistic effects of integration host factor and Fis. J Bacteriol 179: 6367–6377. PubMed: 9335285. 9335285 10.1128/jb.179.20.6367-6377.1997 PMC179552

[7] Walker KA, Atkins CL, Osuna R (1999) Functional determinants of the *Escherichia* *coli* *fis* promoter: roles of −35, −10, and transcription initiation regions in the response to stringent control and growth phase-dependent regulation. J Bacteriol 181: 1269–1280. PubMed: 9973355. 9973355 10.1128/jb.181.4.1269-1280.1999 PMC93506

[8] Ninnemann O, Koch C, Kahmann R (1992) The *E.coli* *fis* promoter is subject to stringent control and autoregulation. EMBO J 11: 1075–1083. PubMed: 1547773. 1547773 10.1002/j.1460-2075.1992.tb05146.x PMC556548

[9] Schneider R, Travers A, Muskhelishvili G (2000) The expression of the *Escherichia* *coli* *fis* gene is strongly dependent on the superhelical density of DNA. Mol Microbiol 38: 167–175. doi:10.1046/j.1365-2958.2000.02129.x. PubMed: 11029698. 11029698

[10] Travers A, Muskhelishvili G (2005) DNA supercoiling — a global transcriptional regulator for enterobacterial growth? Nat Rev Microbiol 3: 157–169. doi:10.1038/nrmicro1088. 15685225

[11] Mallik P, Paul BJ, Rutherford ST, Gourse RL, Osuna R (2006) DksA is required for growth phase-dependent regulation, growth rate-dependent control, and stringent control of *fis* expression in *Escherichia* *coli* . J Bacteriol 188: 5775–5782. doi:10.1128/JB.00276-06. PubMed: 16885445. 16885445 PMC1540068

[12] Osborne SE, Coombes BK (2011) Transcriptional priming of *Salmonella* Pathogenicity Island-2 precedes cellular invasion. PLOS ONE 6: e21648. doi:10.1371/journal.pone.0021648.t001. PubMed: 21738750. 21738750 PMC3125303

[13] Cróinín TÓ, Dorman CJ (2007) Expression of the Fis protein is sustained in late-exponential- and stationary-phase cultures of *Salmonella* *enterica* serovar Typhimurium grown in the absence of aeration. Mol Microbiol 66: 237–251. doi:10.1111/j.1365-2958.2007.05916.x. PubMed: 17784910. 17784910

[14] Faucher SP, Porwollik S, Dozois CM, McClelland M, Daigle F (2006) Transcriptome of *Salmonella* *enterica* serovar Typhi within macrophages revealed through the selective capture of transcribed sequences. Proc Natl Acad Sci U S A 103: 1906–1911. doi:10.1073/pnas.0509183103. PubMed: 16443683. 16443683 PMC1413645

[15] Walker KA (2004) The *Escherichia* *coli* *fis* promoter is regulated by changes in the levels of its transcription initiation nucleotide CTP. J Biol Chem 279: 50818–50828. doi:10.1074/jbc.M406285200. PubMed: 15385561. 15385561

[16] Alexeeva S, Hellingwerf KJ, Teixeira de Mattos MJ (2003) Requirement of Arca for Redox Regulation in *Escherichia* *coli* under Microaerobic but Not Anaerobic or Aerobic Conditions. J Bacteriol 185: 204–209. doi:10.1128/JB.185.1.204-209.2003. PubMed: 12486057. 12486057 PMC141817

[17] Salmon K, Hung S-P, Mekjian K, Baldi P, Hatfield GW et al. (2003) Global gene expression profiling in *Escherichia* *coli* K12. The effects of oxygen availability and FNR. J Biol Chem 278: 29837–29855. doi:10.1074/jbc.M213060200. PubMed: 12754220. 12754220

[18] Dorman CJ (2006) DNA supercoiling and bacterial gene expression. Sci Prog 89: 151–166. doi:10.3184/003685006783238317. PubMed: 17338437. 17338437 PMC10368349

[19] Jin DJ, Cagliero C, Zhou YN (2012) Growth rate regulation in *Escherichia* *coli* . FEMS Microbiol Rev 36: 269–287. doi:10.1111/j.1574-6976.2011.00279.x. PubMed: 21569058. 21569058 PMC3478676

[20] Beach MB, Osuna R (1998) Identification and characterization of the *fis* operon in enteric bacteria. J Bacteriol 180: 5932–5946. PubMed: 9811652. 9811652 10.1128/jb.180.22.5932-5946.1998 PMC107668

[21] Guadarrama-Beltran S (2013) Sustained expression of *fis*, the gene coding for the Fis nucleoid-associated protein, during the stationary phase of growth in *Salmonella* *enterica* . University of Dublin, Trinity College.

[22] Datsenko KA, Wanner BL (2000) One-step inactivation of chromosomal genes in *Escherichia* *coli* K-12 using PCR products. Proc Natl Acad Sci U S A 97: 6640–6645. doi:10.1073/pnas.120163297. PubMed: 10829079. 10829079 PMC18686

[23] Sternberg NL, Maurer R (1991) Bacteriophage-mediated generalized transduction in *Escherichia* *coli* and *Salmonella* *typhimurium* . Methods Enzymol 204: 18–43. doi:10.1016/0076-6879(91)04004-8. PubMed: 1943777. 1943777

[24] Kröger C, Dillon SC, Cameron ADS, Papenfort K, Sivasankaran SK et al. (2012) The transcriptional landscape and small RNAs of *Salmonella* *enterica* serovar Typhimurium. Proc Natl Acad Sci U S A 109: E1277–E1286. doi:10.1073/pnas.1201061109. PubMed: 22538806. 22538806 PMC3356629

[25] Cameron ADS, Dorman CJ (2012) A Fundamental Regulatory Mechanism Operating through OmpR and DNA Topology Controls Expression of *Salmonella* Pathogenicity Islands SPI-1 and SPI-2. PLoS Genet 8: e1002615. doi:10.1371/journal.pgen.1002615.g005. PubMed: 22457642. 22457642 PMC3310775

[26] Cameron ADS, Stoebel DM, Dorman CJ (2011) DNA supercoiling is differentially regulated by environmental factors and FIS in *Escherichia* *coli* and *Salmonella* *enterica* . Mol Microbiol 80: 85–101. doi:10.1111/j.1365-2958.2011.07560.x. PubMed: 21276095. 21276095

[27] Bi C, Benham CJ (2004) WebSIDD: server for predicting stress-induced duplex destabilized (SIDD) sites in superhelical DNA. Bioinformatics 20: 1477–1479. doi:10.1093/bioinformatics/bth304. PubMed: 15130924. 15130924

[28] Argaman L, Hershberg R, Vogel J, Bejerano G, Wagner EG et al. (2001) Novel small RNA-encoding genes in the intergenic regions of *Escherichia* *coli* . Curr Biol 11: 941–950. doi:10.1016/S0960-9822(01)00270-6. PubMed: 11448770. 11448770

[29] Mika F, Hengge R (2005) A two-component phosphotransfer network involving ArcB, ArcA, and RssB coordinates synthesis and proteolysis of σS (RpoS) in *E.* *coli* . Genes Dev 19: 2770–2781. doi:10.1101/gad.353705. PubMed: 16291649. 16291649 PMC1283968

[30] Wang H, Noordewier M, Benham CJ (2004) Stress-induced DNA duplex destabilization (SIDD) in the *E.* *coli* genome: SIDD sites are closely associated with promoters. Genome Res 14: 1575–1584. doi:10.1101/gr.2080004. PubMed: 15289476. 15289476 PMC509266

[31] Farewell A, Kvint K, Nyström T (1998) Negative regulation by RpoS: a case of sigma factor competition. Mol Microbiol 29: 1039–1051. doi:10.1046/j.1365-2958.1998.00990.x. PubMed: 9767572. 9767572

[32] Dorman CJ, Barr GC, Ni Bhriain N, Higgins CF (1988) DNA supercoiling and the anaerobic and growth phase regulation of *tonB* gene expression. J Bacteriol 170: 2816–2826. PubMed: 2836373. 2836373 10.1128/jb.170.6.2816-2826.1988 PMC211208

[33] Hsieh LS, Burger RM, Drlica K (1991) Bacterial DNA supercoiling and [ATP]/[ADP]. Changes associated with a transition to anaerobic growth. J Mol Biol 219: 443–450. doi:10.1016/0022-2836(91)90185-9. PubMed: 1646892. 1646892

[34] Berthoumieux S, de Jong H, Baptist G, Pinel C, Ranquet C et al. (2013) Shared control of gene expression in bacteria by transcription factors and global physiology of the cell. Mol Syst Biol 9: 1–11. doi:10.1038/msb.2012.70. PubMed: 23340840. PMC3564261 23340840

[35] Keane OM, Dorman CJ (2003) The *gyr* genes of Salmonella enterica serovar Typhimurium are repressed by the factor for inversion stimulation, Fis. Mol Genet Genomics 270: 56–65. doi:10.1007/s00438-003-0896-1. PubMed: 12898222. 12898222

[36] Maciag A, Peano C, Pietrelli A, Egli T, De Bellis G et al. (2011) *In* *vitro* transcription profiling of the σ^S^ subunit of bacterial RNA polymerase: re-definition of the σ^S^ regulon and identification of σ^S^-specific promoter sequence elements. Nucleic Acids Res 39: 5338–5355. doi:10.1093/nar/gkr129. PubMed: 21398637. 21398637 PMC3141248

[37] Bordes P, Conter A, Morales V, Bouvier J, Kolb A et al. (2003) DNA supercoiling contributes to disconnect σ^S^ accumulation from σ^S^-dependent transcription in *Escherichia* *coli* . Mol Microbiol 48: 561–571. doi:10.1046/j.1365-2958.2003.03461.x. PubMed: 12675812. 12675812

[38] Grainger DC, Goldberg MD, Lee DJ, Busby SJW (2008) Selective repression by Fis and H-NS at the *Escherichia* *coli* *dps* promoter. Mol Microbiol 68: 1366–1377. doi:10.1111/j.1365-2958.2008.06253.x. PubMed: 18452510. 18452510

[39] Mallik P, Pratt TS, Beach MB, Bradley MD, Undamatla J et al. (2004) Growth phase-dependent regulation and stringent control of *fis* are conserved processes in enteric bacteria and involve a single promoter (*fis* P) in *Escherichia* *coli* . J Bacteriol 186: 122–135. doi:10.1128/JB.186.1.122-135.2004. PubMed: 14679232. 14679232 PMC303451

[40] Zheng D (2004) Identification of the CRP regulon using in vitro and in vivo transcriptional profiling. Nucleic Acids Res 32: 5874–5893. doi:10.1093/nar/gkh908. PubMed: 15520470. 15520470 PMC528793

