# Supplementary figures and images for: Transmission of an Oxygen Availability Signal at the Salmonella enterica Serovar Typhimurium fis Promoter
Source: PLoS One. 2013 Dec 16;8(12):e84382. doi: 10.1371/journal.pone.0084382 (PMC3865300; doi:10.1371/journal.pone.0084382)

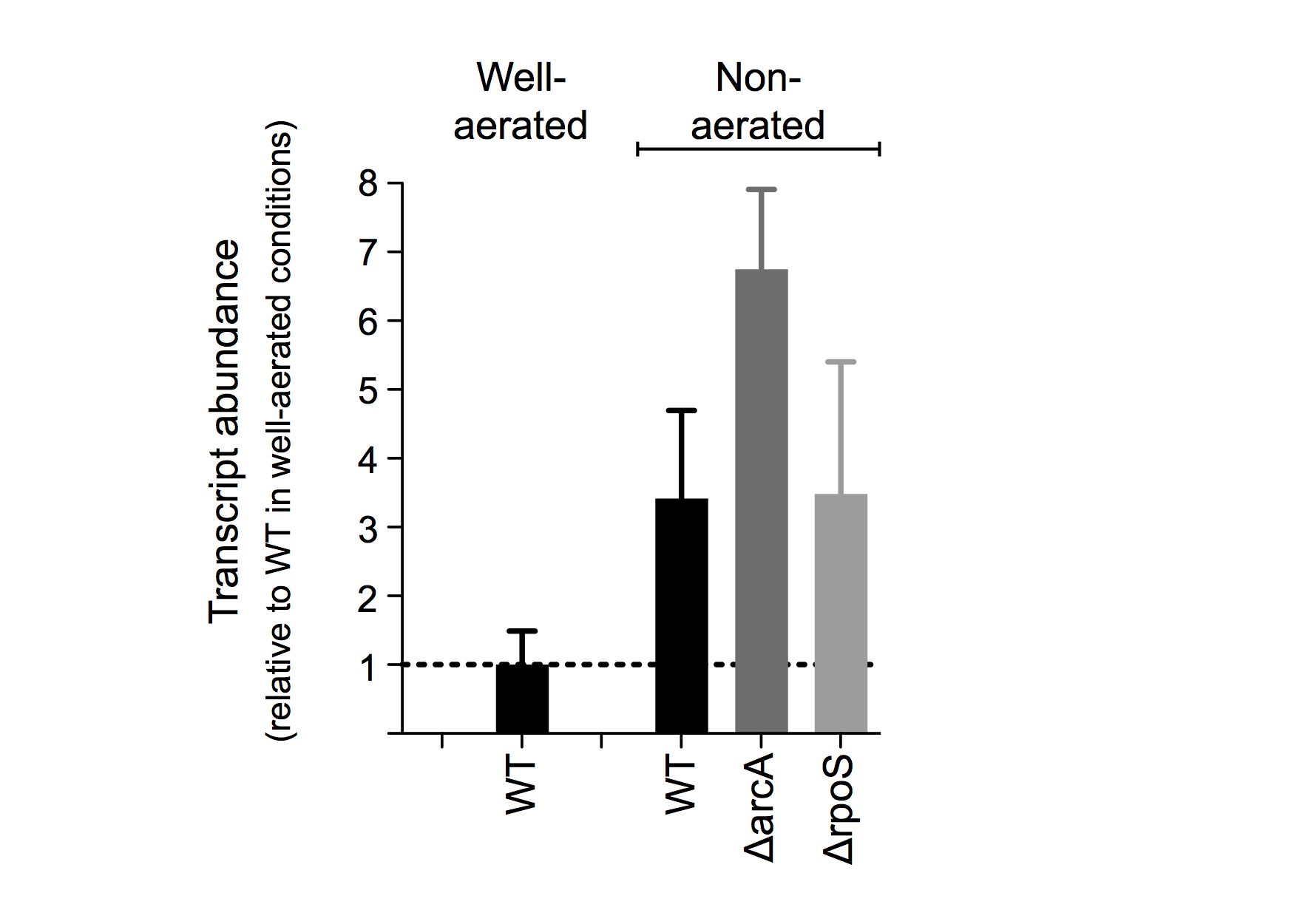

Supplement: Figure S1 — Quantitative PCR measurement of fis transcript in mutants. Total (Pfis-1 plus Pfis-2) fis transcript abundance, expressed relative to wild type at 22 hours in well-aerated conditions. (TIFF) [file pone.0084382.s001.tiff]
