# Supplementary material for: Transmission of an Oxygen Availability Signal at the Salmonella enterica Serovar Typhimurium fis Promoter
Source: PLoS One. 2013 Dec 16;8(12):e84382. doi: 10.1371/journal.pone.0084382 (PMC3865300; doi:10.1371/journal.pone.0084382)
Supplement: Table S1 — Bacterial strains and plasmids used in this study. (DOCX) [file pone.0084382.s002.docx]

Table S1: Bacterial strains and plasmids used in this study

| Strain or plasmid | Description and relevant genotype | Reference |
| --- | --- | --- |
| S. enterica serovar Typhimurium | | |
| SL1344 | rpsL hisG | [41] |
| SL1344 fis-gfp^TCD^ | fis-gfp^TCD^; the Kan^R^ marker was removed | This study |
| SL1344 ΔarcA | arcA::kan, Kan^R^ | This study |
| SL1344 ΔarcAΔrpoS | fis-gfp^TCD^, arcA::kan, rpoS::cat, Kan^R^, Cm^R^ | This study |
| SL1344 Δfnr | fnr::kan, Kan^R^ | This study |
| SL1344 Δcrp | crp::cat, Cm^R^ | This study |
| SL1344 Δcya | cya::Tn10, | This study |
| SL1344 ΔrpoS | rpoS::kan, Kan^R^ | [41] |
| SL1344 Δfis | fis::kan, Kan^R^ | [25] |
| Plasmids | | |
| pCoC2 | gfp^TCD^::kan, Kan^R^ | [42] |
| pZec | gfp+, ColE1 replication origin, Carb^R^ | [25] |
| pZec-Pfis (−298) | Pfis to position -298 cloned in pZec, Carb^R^ | This study |
| pZec-Pfis (−198) | Pfis to position -198 cloned in pZec, Carb^R^ | This study |
| pZec-Pfis (−49) | Pfis to position --49 cloned in pZec, Carb^R^ | This study |
|  | | |
| Carb^R^, carbenicillin resistant; Cm^R^, chorlamphenicol resistant; Kan^R^, kanamycin resistant | | |

41. Hoiseth SK, Stocker BA (1981) Aromatic-dependent *Salmonella typhimurium* are non-virulent and effective as live vaccines. Nature 291: 238–239.

42. Kowarz L, Coynault C, Robbe-Saule V, Norel F (1994) The *Salmonella typhimurium katF* (*rpoS*) gene: cloning, nucleotide sequence, and regulation of *spvR* and *spvABCD* virulence plasmid genes. J Bacteriol 176: 6852–6860.

43. Corcoran CP, Cameron ADS, Dorman CJ (2010) H-NS silences *gfp*, the Green Fluorescent Protein gene: *gfp*^TCD^ is a genetically remastered *gfp* gene with reduced susceptibility to H-NS-mediated transcription silencing and with enhanced translation. J Bacteriol 192: 4790–4793. doi:10.1128/JB.00531-10.
