# Supplementary material for: Transmission of an Oxygen Availability Signal at the Salmonella enterica Serovar Typhimurium fis Promoter
Source: PLoS One. 2013 Dec 16;8(12):e84382. doi: 10.1371/journal.pone.0084382 (PMC3865300; doi:10.1371/journal.pone.0084382)
Supplement: Table S2 — Oligonucleotide primers used in this study. (DOCX) [file pone.0084382.s003.docx]

Table S2: Oligonucleotide primers used in this study

| Primer Name | Sequence |
| --- | --- |
| Mutant construction | |
| Se.delta.arcA-F | 5'- ACT TCC TGT TTC GAT TTA GTT GGC AAT TTA GGT AGC AAA CGT GTA GGC TGG AGC TGC TTC -3' |
| Se.delta.arcA-R | 5'- AAC TTA CCG GCT GTT TTT ACA GTT TGG CGC CTG GGC CGA ACA TAT GAA TAT CCT CCT TA -3' |
| Se.delta.fnr-F | 5'- CTT CTC CGG GAT AGC TCA GAC TTA CGC GCT CAC CAA AAA GGT GTA GGC TGG AGC TGC TTC -3' |
| Se.delta.fnr-R | 5'- ACG ATA TGG CAG AAG ATA ACA TCA ATG GTT TAG CTG ACG TCA TAT GAA TAT CCT CCT TA -3' |
| Se.delta.crp-F | 5'- TCT GGC TCT GGA GAC AGC TTA TAA CAG AGG ATA ACC GCG CGT GTA GGC TGG AGC TGC TTC -3' |
| Se.delta.crp-R | 5'- ACA AAA ATG GCG CAT GAT AAA ACG CGC CAT TCT GAC GGA ACA TAT GAA TAT CCT CCT TA -3' |
| Se.fis.gfpTCD-For | 5'- GCG TAA AAA ATT AAA AAA ATA CGG CAT GAA CTA ATT TCG AAG GAG GAA TTC ACC ATG AGC AAA GGC GAA GAG CT -3' |
| Se.fis.gfpTCD-Rev | 5'- CCA TGC CGA GTA GCG CCT TTT TAA ACA AGC AGT TAG CTA ACA TAT GAA TAT CCT CCT TA -3' |
| Quantitative PCR and 5′ RACE | |
| Pfis.anaerTSS.RT.For | 5'- CCG CTT ATC TTG TTT CTG GAC GAA G -3' |
| Pfis.anaerTSS.RT.Rev | 5'- TGC ACG AGA TGA AAG GCC AAA C -3' |
| Pfis.expTSS.RT.For | 5'- TCA TGC GCA TCG GAC AAT ATC AGC -3' |
| Pfis.expTSS.RT.Rev | 5'- ATC TCA TAG CAC AGC GTC CGG AAA -3' |
| Promoter truncation by inverse PCR | |
| PfisS.trunc(-196).EcoRV-For | 5'- GAT ATC TGT TTC TGG ACG AAG AAA TA-3' |
| PfisS.trunc(-47).SacI-For | 5'- GAG CTC TCA AAG TTT GGC CTT TCA TC-3' |
| pZep.trunc-Rev | 5'- ATT TGT CCT ACT CAG GAG AG-3' |
